# Effect of Home-Based Virtual Reality Training on Upper Extremity Recovery in Patients With Stroke: Systematic Review

**DOI:** 10.2196/69003

**Published:** 2025-04-04

**Authors:** Jiaqi Huang, Yixi Wei, Ping Zhou, Xiaokuo He, Hai Li, Xijun Wei

**Affiliations:** 1 Rehabilitation Lab of Mix Reality Shenzhen Hospital Southern Medical University Shenzhen China; 2 Department of Rehabilitation Medicine The Fifth Hospital of Xiamen City Xiamen China; 3 Department of Gynecology Shenzhen Hospital Southern Medical University Shenzhen China; 4 Department of Rehabilitation Medicine Shenzhen Hospital Southern Medical University Shenzhen China; 5 Department of Occupational Therapy School of Rehabilitation Sciences Southern Medical University Shenzhen China

**Keywords:** stroke, upper extremity, virtual reality, home-based, rehabilitation, upper extremity, motor control, recovery

## Abstract

**Background:**

Stroke is a leading cause of long-term disability, often resulting in upper extremity dysfunction. Traditional rehabilitation methods often face challenges such as limited patient access to resources and lack of sustained motivation. Home-based virtual reality (VR) training is gaining traction as an innovative, sustainable, and interactive alternative. However, the effect of home-based VR, specifically for upper extremity recovery in patients with stroke, remains insufficiently explored.

**Objective:**

This systematic review aims to synthesize existing evidence to evaluate the impact of home-based VR interventions on upper extremity function recovery in patients with stroke.

**Methods:**

This systematic review followed the guidelines of the PRISMA (Preferred Reporting Items for Systematic Reviews and Meta-Analyses). A comprehensive literature search was conducted across PubMed, Web of Science, Scopus, and CINAHL (Cumulative Index to Nursing and Allied Health Literature) Ultimate databases, targeting English-language randomized controlled trials (RCTs) published up to June 30, 2024. Eligible studies involved patients with stroke with upper extremity dysfunction who received home-based VR interventions. Data extraction was performed by 2 independent reviewers, and study quality was assessed using the Physiotherapy Evidence Database scale. Due to heterogeneity in study designs and outcome measures, a narrative synthesis was performed instead of a meta-analysis.

**Results:**

A total of 8 RCTs with 392 participants were included. This review shows that home-based VR training positively affects upper extremity function recovery in patients with stroke, especially in motor control improvement. Customized VR systems were more effective than commercial VR systems in patients with moderate to severe disorders. Although studies generally showed positive results, differences in intervention protocols and small sample sizes limited the validity of results. The effect of VR therapy also varied based on the VR system type, intervention intensity, and the functional level of patients.

**Conclusions:**

This systematic review shows that home-based VR training has a positive impact on upper extremity rehabilitation for patients with stroke, particularly in those with varying degrees of dysfunction. However, heterogeneity in study design and differences in outcome measures affect the reliability of the current conclusions. Future studies should include larger, standardized RCTs with long-term follow-up to assess their continued effects. Future research should explore how VR technology can be integrated into comprehensive rehabilitation programs, focusing on patient-centered approaches that incorporate sustainable, personalized technology, and support services to optimize recovery outcomes.

**Trial Registration:**

PROSPERO CRD42024526650; https://tinyurl.com/5dny5bhp

## Introduction

Stroke is a leading cause of disability worldwide, frequently resulting in upper extremity impairments that significantly limit daily function [1]. Studies have shown that approximately 80% of stroke survivors experience upper extremity dysfunction, severely limiting their ability to perform daily activities independently and affecting their overall health [2]. Traditional rehabilitation approaches, such as physical therapy and occupational therapy, have been the mainstay of treatment for poststroke upper extremity recovery. However, these methods often face limitations, including the availability of trained therapists, high costs, and the lack of engaging and motivating exercises for patients [3]. These challenges become even more pronounced in home-based settings, where supervision and resources are often more limited [4].

In home-based settings, routine care often lacks sufficient intensity and task-specific training, which are critical for motor learning and functional recovery [3]. Similarly, traditional therapies at home may suffer from limited professional supervision, reduced patient motivation, and lower adherence rates compared to hospital-based programs [5,6]. These limitations hinder the effectiveness of home-based interventions and highlight the need for alternative approaches that can overcome these barriers, such as virtual reality (VR) training [4,7]. In recent years, home-based VR training has emerged as a potential alternative for rehabilitation in patients with stroke [7,8]. VR technology enables patients to perform repetitive, task-specific movements in an engaging, interactive environment, potentially enhancing motor recovery, and improving adherence to rehabilitation protocols [9].

VR technology has garnered significant attention for its potential to enhance rehabilitation outcomes across various treatment environments. Research indicates that VR-based therapies, when used as an adjunct to conventional rehabilitation methods, can significantly enhance somatic functional recovery in patients with stroke [10-14]. For instance, Laver et al [5] noted in their systematic review that rehabilitation training integrating VR in hospital settings can improve upper extremity function and overall recovery. In community settings, Kim et al [15] confirmed that VR training effectively enhances balance in patients with stroke. Furthermore, Hao et al [16] explored the impact of home-based VR rehabilitation and found that this method significantly improves upper extremity mobility and walking ability in patients. These studies suggest that incorporating VR technology into traditional therapies can provide targeted and repetitive practice, crucial for promoting neuroplasticity and motor learning. These trials suggest that home-based VR could be a promising and effective method for upper extremity training in stroke rehabilitation.

However, existing research shows varied effects of VR training systems on upper extremity function, with differences particularly evident between commercial and customized VR systems. Commercial VR systems, designed primarily for general consumer use, are often marketed for gaming and entertainment purposes rather than rehabilitation [17]. While these systems can be cost-effective and engaging, they frequently lack the specific therapeutic features needed for rehabilitation, resulting in inconsistent outcomes [18]. In contrast, customized VR systems are specifically developed for clinical or rehabilitative applications. These systems are tailored to meet individual therapeutic needs, offering features such as personalized exercises, real-time performance feedback, and adaptive difficulty levels [19]. These characteristics make them especially effective for patients with severe impairments [20]. Despite their promise, discrepancies in outcomes between these 2 VR system types underscore the need for further investigation into how each can be optimally used for different patient populations.

Furthermore, the evidence supporting the effect of home-based VR training for upper extremity recovery in patients with stroke is still limited and remains to be systematically evaluated. A recent review investigated VR as an approach to telerehabilitation, and the results showed that VR played a positive role in promoting upper extremity function [16]. Nevertheless, upper extremity function was not the main focus of their research interests, and upper extremity assessment was not used in some of the included studies. Nor is the home setting the only study setting, which also includes small clinics and community rehabilitation. Consequently, the heterogeneity of the study setting, methods, and assessments limits the strength of the evidence.

Despite the growing interest in home-based VR training for upper extremity recovery in patients with stroke, there is a lack of comprehensive and up-to-date evidence synthesis on this topic. To address these gaps, this systematic review aims to comprehensively evaluate the effect, quality, and intervention characteristics of home-based VR training as reported in existing systematic reviews and randomized controlled trials (RCTs). By analyzing key factors such as the population studied, the duration and intensity of interventions, and the type of VR technology used, this review seeks to identify elements that influence rehabilitation outcomes and provide a clearer understanding of how home-based VR training can be optimized for stroke rehabilitation.

## Methods

### Study Design and Registration

This systematic review was conducted and reported according to the guidelines of the PRISMA (Preferred Reporting Items for Systematic Reviews and Meta-Analysis) [21] (Multimedia Appendix 1). As per the PRISMA guidelines, the protocol is registered with the PROSPERO (International Prospective Register of Systematic Reviews) (registration number CRD42024526650).

### Search Strategy

This systematic review searched the English articles from PubMed, Web of Science, Scopus, and CINAHL Ultimate databases from inception to June 30, 2024. Search terms include (virtual reality OR VR OR video game OR telerehabilitation OR gamification OR exergame OR virtual environment) AND stroke AND upper extremity AND (home OR community). The search strategies are presented in the Multimedia Appendix 2.

### Eligibility Criteria

#### Study Types

This systematic review aims to describe and qualitatively assess the effect of home-based VR training on upper extremity recovery in patients with stroke. To ensure high-quality evidence, we included only RCTs, defined as studies that used a random allocation process to assign participants to intervention or control groups. Quasi-randomized trials or nonrandomized studies were excluded.

#### Inclusion and Exclusion Criteria

Studies that met the following criteria were included: (1) all participants were diagnosed with stroke, (2) all participants presented upper extremity dysfunction, (3) the interventions were implemented in the home environment, (4) the experimental group used VR as the primary intervention method, and (5) upper extremity performance was assessed as the primary outcome measure. Exclusion criteria are as follows: (1) the participants had neurological diseases other than stroke; (2) the study type was a nonrandomized controlled trial, such as quasi-randomized or observational studies; (3) articles published in a non-English journal; and (4) articles without full text or peer-reviewed publication status.

#### Participants

This review included patients diagnosed with stroke and presenting upper extremity dysfunction. Stroke was defined as a neurological deficit resulting from an acute vascular event in the central nervous system (ie, brain, retina, or spinal cord) with a vascular cause, typically diagnosed through clinical evaluation and neuroimaging, including computed tomography or magnetic resonance imaging [22]. Upper extremity dysfunction was defined as any impairment in arm or hand function following a stroke, regardless of severity.

#### Types of Interventions

The experimental group used VR technology for home rehabilitation training for upper extremity function of patients with stroke. VR is a simulation environment created by computer technology so that users can experience and interact with it. VR systems can be immersive, semi-immersive, or nonimmersive, and both commercial and customized systems are eligible.

#### Comparisons or Control

The review included studies with a comparator, which can be any of the following: traditional treatment, routine care, or another form of VR in a hospital, clinic, or home setting.

### Outcome Measures

This review included changes in patients’ upper extremity function as the primary outcome, using a variety of assessment tools to evaluate changes in upper extremity function, including but not limited to Fugl-Meyer Assessment upper extremity section (FMA-UE), Action Research Arm Test (ARAT), Wolf Motor Function Test (WMFT), Box and Block Test (BBT), Nine‐Hole Peg Test (NHPT), Purdue Peg Test (PPT) These assessments measured improvements in motor function from baseline to the end of the follow-up period.

### Data Collection and Analysis

#### Selection of Studies

In total, 2 reviewers independently collated and uploaded the data to EndNote 21 (Clarivate Analytics), removed duplicate data, and screened all titles and abstracts to determine whether they met the inclusion criteria for this review. Finally, the full text of eligible studies was evaluated in detail according to the inclusion criteria. The 2 reviewers searched 4 databases for relevant reviews on similar topics. They performed citation searches and full-text evaluations of the references to these reviews to identify other eligible studies. Any conflicts during this process will be discussed and resolved in team meetings.

#### Data Extraction and Management

In total, two reviewers independently extracted data from included articles, including the following items: (1) author and year of publication, (2) mean age, (3) mean time since stroke, (4) inclusion and exclusion criteria, (5) the number of participants, (6) intervention intensity and frequency, (7) description of intervention measures, (8) measurement time points, (9) upper extremity function baseline, (10) primary outcome measurement, (11) secondary outcome measures, and (12) major findings. A total of 2 reviewers cross-check the extracted data, and disagreements are discussed and resolved in a team meeting. All data is collated, archived, and stored in a secure electronic database.

#### Qualitative Synthesis

Due to the heterogeneity of the included studies (eg, differences in VR system types, patient populations, and outcome measures), a meta-analysis was not feasible. Therefore, a qualitative synthesis was performed to summarize and interpret the findings. Given the small number of studies using commercial VR (n=1) and focusing on participants with moderate upper extremity dysfunction (n=1), subgroup analyses were not conducted. The narrative synthesis focused on the following.

### Key Findings

We summarized the improvements in upper extremity function, with common trends observed across studies, particularly in motor control and patient engagement.

### Variations

#### Overview

Differences in intervention design, outcome measures, and patient characteristics were discussed, noting how these factors may have influenced the results.

#### Assessment of Methodological Quality

To determine the validity of the included articles, we used the Physiotherapy Evidence Database scale (PEDro scale) to assess the included RCTs [23,24]. The PEDro scale consists of eleven items: (1) eligibility criteria and source of participants, (2) random allocation, (3) concealed allocation, (4) baseline comparability, (5) blinding of participants, (6) blinding of therapists, (7) blinding of assessors, (8) less than 15% dropouts, (9) intention-to-treat analysis, (10) between-group statistical comparisons, and (11) point and variability measures. The researchers considered that scores 9-10 are excellent quality, 6-8 are good quality, 4-5 are fair quality, and <4 are regarded as poor quality. In total, 2 independent reviewers assessed the risk of bias for each study, and differences were resolved through discussion with a third reviewer. The results of this assessment were incorporated into the interpretation of findings, with higher-quality studies given greater weight in the synthesis.

## Results

### Study Identification

**Figure 1:**
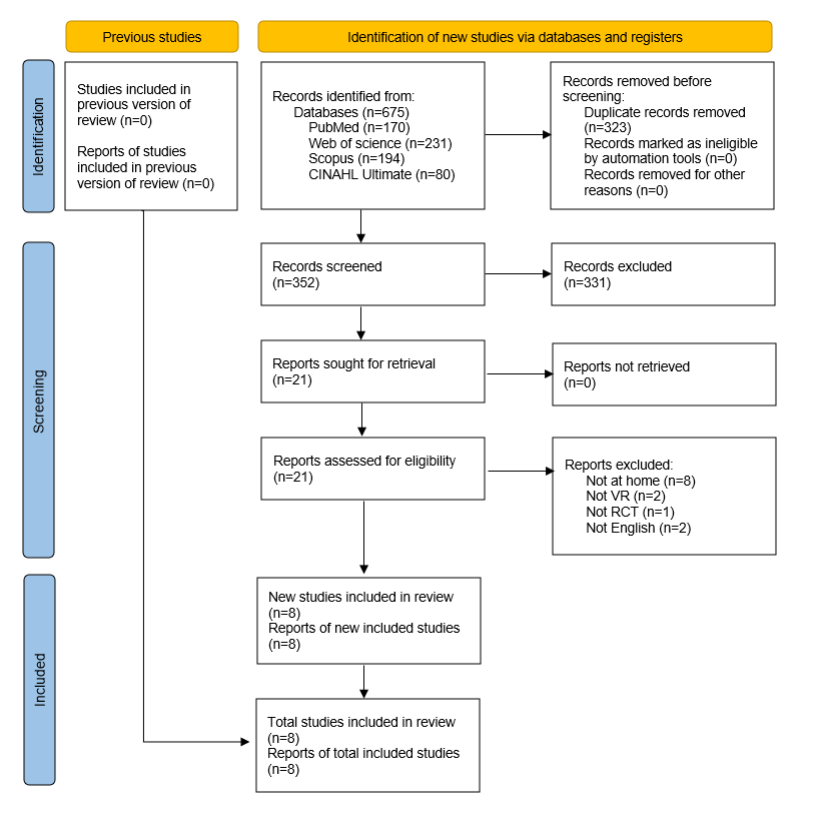
PRISMA (Preferred Reporting Items for Systematic Reviews and Meta-Analysis) 2020 flow diagram for this systematic review. CINAHL: Cumulative Index to Nursing and Allied Health Literature; RCT: randomized controlled trial; VR: virtual reality.

A total of 675 articles were searched in the electronic database (PubMed: n=170, Web of Science: n=231, Scopus: n=194, and CINAHL Ultimate: n=80). After removing duplicates and 2 screening phases, 8 RCTs qualified for inclusion in this review. The literature retrieval and screening process is shown in Figure 1.

### Study Characteristics

In total, 8 randomized controlled trials were published from 2017 to 2023 and included 392 participants, with one study having a big sample size of 235 and the rest ranging from 11 to 39 (Table 1). In 8 of the included studies, participants were diagnosed with stroke and upper extremity dysfunction. The studies varied in terms of participant characteristics, with most focusing on patients with mild to moderate upper extremity dysfunction. Based on the baseline data from various assessment tools, we found that 4 studies (FMA-UE, ARAT) reported moderate impairment, with baseline scores ranging from 40 to 45 points [25-28]. A total of 2 studies using the WMFT and BBT tests indicated mild functional impairment, with baseline scores generally around 2-3 seconds for WMFT [29,30]. In addition, one study (Hernandez et al [31]) indicated that some participants in the experimental group had a severe impairment, with baseline FMA-UE scores below 30 [31]. Although one study did not conduct baseline assessments, based on the Chedoke-McMaster scoring (with a 2-6 range for inclusion criteria), it can be inferred that the participants in this study had moderate functional impairment at baseline [32].

The duration and frequency of home-based VR interventions varied across the studies, typically lasting between 4 and 8 weeks, with longer durations showing more sustained improvements in upper extremity function. Thielbar et al [26] found that a 2-week, high-intensity, daily 1-hour intervention with a multimodal approach improved function significantly more than a single-modality intervention, highlighting the importance of adaptable interventions for maintaining patient engagement. Similarly, Allegue et al [32] found that moderate-intensity interventions (30 min, 5 times a wk) for 3 months led to significant improvements in FMA-UE and MAL, underscoring the value of structured, long-term interventions. Wilson et al [29] showed that a flexible 8-week intervention with the EDNA system (3-4 sessions per wk) significantly improved upper extremity function, emphasizing the role of consistent engagement. Finally, Standen et al [30] demonstrated that even lower-intensity training (20 min, 3 times a wk) for 8 weeks effectively improved function, supporting the importance of adaptable intervention strategies. Overall, the studies suggest that structured yet adaptable interventions can maintain patient engagement and maximize functional gains in upper extremity rehabilitation.

In the included studies, VR technology types cover commercial and custom systems, each with unique advantages and application scenarios. Commercial systems such as the Nintendo Wii are inexpensive, easy to use, and stimulating for patients, but have limitations regarding rehabilitation design and personalized training [27]. Customized systems such as Jintronix, EDNA-22, and Glove Rehabilitation Application for Stroke Patients (GRASP) provide personalized training tasks and detailed feedback that can be adjusted to the patient’s functional needs, significantly improving training results [25,29-31]. In addition, some studies incorporated telehealth technology into VR systems (eg, GRASP and VirTele), improving the feasibility and adherence to home-based rehabilitation [25,32]. Multiuser VR systems, such as Virtual Environment for Rehabilitative Gaming Exercises (VERGE), introduced interactive and social components that increased participation and user motivation [26]. These diverse VR technologies provide effective rehabilitation options for patients with different levels of impairment, showcasing their potential for remote and personalized rehabilitation.

**Table 1:** Characteristics of included studies.

Author	Participants	Sample size	Intervention	Groups	Measure	Primary outcomes	Secondary outcomes	Findings
Adams et al [25]	Shoulder flexion, abduction, adduction ≥30°; Shoulder rotation ≥15°; Elbow flexion ≥45°.	21	45 min 4 d/wk 8 wk	EG^a^: GRASP^b^ HEP^c^ CG^d^: UCT^e^	T0: 0 T1: 8 wk	FMA-UE^f^	WMFT^g^ BBT^h^ MAL^i^	Both groups improved significantly on the FMUE and MAL; Significant difference between groups only in the FMUA and MAL; EG showed better performance than CG.
Allegue et al [32]	CAHAI^j^ 2-6; No longer receiving rehabilitation services and were able to use the exergame system.	11	30 min 5 d/wk 3 mo	EG: Jintronix CG: GRASP	T0: 0 T1: 3 mo Follow-up: 1 and 2 mo	FMA-UE	MAL SIS-16^k^ TSRQ-15^l^	Both groups showed improvement in all outcome measures; No significant difference in all outcomes between groups; Both groups maintained improved outcomes only on FMUE and MAL at follow-up.
Hernandez et al [31]	First-time stroke; Onset time >6 mo; CAHAI 2-6; No longer receiving rehabilitation services.	19	20 min 5 d/wk 4 wk	EG: Jintronix CG: GRASP	T0: 0 T1: 4 wk Follow-up: 4 wk	FMA-UE	SIS^m^ MAL	Both groups improved significantly on the FMA-UE, but most disappeared during follow-up; No significant differences in all outcomes between groups.
Wilson et al [29]	Age＞18 y; Shoulder flexion ≥20°; Elbow flexion ≥90°; The ability to maintain the wrist in a neutral position while holding an object used by the EDNA^n^ system.	19	30 min 3~4 d/wk 8 wk	EG: EDNA+TAU^o^ CG: GRASP+TAU	T0: 0 T1: 8 wk Follow-up: 3 mo	BBT MoCa^p^	9-HPT^q^ SIS NFI^r^	EG groups improved significant on the BBT, and moderate (but nonsignificant) improvement on the 9-HPT, SIS, and NFI. Significant difference between groups in the BBT and MoCa, EG better performance than CG.
Thielbar et al [26]	Stroke ＞6 mo; CAHAI 3-5	21	1 h/d 4 d/wk 2 wk MU^s^ 2 wk SU^t^	EG: VERGE^u^ MU CG: VERGE SU	T0: 0 T1: 2 wk T3: 4 wk	Time and displacement recorded by the VERGE system.	FMA-UE	Both groups improved in arm displacement and training time, but EG improved significantly; All groups improved significant on the FMA-UE; Both groups showed greater mean improvement during the MU portion, although this difference was not significant.
Adie et al [27]	Stroke＜6 mo; MRC^v^＜5.	235	45 min/d 6wk	EG: Wii CG: GRASP	T0: 0 T1: 6 wk T2: 6 mo	ARAT^w^	COPM^x^ SIS MRS^y^ EQ-5D^z^ 3L	Both groups improved significant on the ARAT; No significant difference in all outcomes between groups.
Ballester et al [28]	First-ever stroke >12 mo; MRC＞2; Age between 45-85 y; Previous experience with RGS^aa^ in the clinic.	39	20 min 5 d/wk 3 wk	EG: RGS CG: OT^ab^	T0: 0 T1: 15 d T3: 12 wk	FMA-UE CAHAI	BI^ac^ Asp^ad^ MRC	EG groups improved significant on the CAHAI.
Standen et al [30]	Age＞18 y; No longer receiving rehabilitation services; Patients were excluded if they had no detectable movement in the arm.	27	20 min 3 times/d 8 wk	EG: Virtual glove CG: Usual care	T0: 0 T1: 4 wk after T0 T2: 8 wk	WMFT	9-HPT MAL NEADL^ae^	EG groups improved significant on the WMFT and MAL at midpoint.

^a^EG: experimental group.

^b^GRASP: Glove Rehabilitation Application for Stroke Patients.

^c^HEP: home exercise program.

^d^CG: control group.

^e^UCT: usual and customary care.

^f^FMA-UE: Fugl-Meyer Assessment upper extremity.

^g^WMFT: Wolf Motor Function Test.

^h^BBT: Box and Blocks Test.

^i^MAL: Motor Activity Log.

^j^CAHAI: Chedoke Arm and Hand Activity Inventory.

^k^SIS-16: Stroke Impact Scale-16.

^l^TSRQ-15: Treatment Self-Regulation Questionnaire-15.

^m^SIS: Stroke Impact Scale.

^n^EDNA: Elements by Dynamic Neural Arts.

^o^TAU: treatment as usual.

^p^MoCa: Montreal Cognitive Assessment.

^q^9-HPT: Nine-Hole Peg Test.

^r^NFI: Neurobehavioural Function Inventory.

^s^MU: multiuser.

^t^SU: single-user.

^u^VERGE: Virtual Environment for Rehabilitative Gaming Exercises.

^v^MRC: Medical Research Council scale.

^w^ARAT: Action Research Arm Test.

^x^COPM: Canadian Occupational Performance Measure.

^y^MRS: Modified Rankin Scale.

^z^EQ-5D 3L: Quality of life measure.

^aa^RGS: Rehabilitation Gaming System.

^ab^OT: occupational therapy.

^ac^BI: Barthel Index.

^ad^Asp: Ashworth Scale Proximal limb.

^ae^NEADL: Nottingham Extended Activities of Daily Living.

### Effect of Home-Based VR Training

Numerous studies have demonstrated the positive impact of home-based VR training on improving upper extremity function in patients with stroke. For instance, Adams et al [25] found that the GRASP HEP system significantly improved FMUE by 10.1 points (*P*<.001). Similarly, Allegue et al [32] showed significant improvements in FMA-UE using the VirTele system in comparison to the GRASP system in the control group. In addition, Wilson et al [29] demonstrated that the EDNA-22 system also outperformed traditional rehabilitation methods in improving upper extremity function, with a mean improvement of 11.2 blocks (*P*=.04) on the BBT. These findings collectively underscore the effectiveness of customized VR systems in promoting upper extremity rehabilitation.

Several studies revealed significant differences between intervention and control groups in terms of upper extremity function. In Adams et al [25], the GRASP HEP group demonstrated a significant 8.6-point improvement in FMUE compared to the UCT (usual and customary care) group (*P*=.002). Furthermore, in Thielbar et al [26], participants in the MU VR mode had a significantly higher arm displacement (414.6 m, *P*=.02) than those in the SU VR mode (327 m), showing the greater benefits of interactive VR systems. On the other hand, Adie et al. (2017) reported no significant difference between the Wii system (commercial VR) and the control group in ARAT (*P*>.05). These results suggest that while home-based VR training is generally effective, tailored VR systems tend to produce more significant improvements compared to traditional methods or commercial VR systems.

PEDro scale showed 4 studies scored 7; three studies scored 6, indicating these studies were of good quality methodologically. Only one study showed relatively low quality, but it reached fair (PEDro scale=5). Common limitations across the studies included the lack of blinding of participants and therapists. Detailed information on the risk of bias is presented in Table 2.

**Table 2:** Physiotherapy Evidence Database scores of included studies.

Studies	Items^a^												Score (0-10)
	1	2	3	4	5	6	7	8	9	10	11		
Adams et al [25]	✓	✓	✓	✓			✓	✓		✓	✓	7	
Allegue et al [32]	✓	✓	✓	✓							✓	4	
Hernandez et al [31]	✓	✓	✓	✓			✓	✓		✓	✓	7	
Wilson et al [29]	✓	✓	✓	✓			✓	✓		✓	✓	7	
Thielbar et al [26]	✓	✓	✓	✓				✓		✓	✓	6	
Adie et al [27]	✓	✓	✓	✓			✓	✓		✓	✓	7	
Ballester et al [28]	✓	✓	✓	✓				✓		✓	✓	6	
Standen et al [30]	✓	✓	✓	✓			✓			✓	✓	6	

^a^Rating items: 1: eligibility criteria and source of participants (item 1 evaluates external validity and does not contribute to the total score), 2: random allocation, 3: concealed allocation. 4: baseline comparability, 5: blinded participants, 6: blinded therapists, 7: blind assessors, 8: adequate follow-up, 9: intention-to-treat analysis, 10: between-group comparisons, 11: point estimates and variability.

## Discussion

### Principal Findings

This systematic review aimed to evaluate the impact of home-based VR training on upper extremity function recovery after stroke. The results generally suggest that home-based VR interventions have a positive role in promoting upper extremity function recovery. However, several factors such as patient characteristics, VR system types, intervention intensity, and study heterogeneity need to be considered to fully understand the treatment effects.

### Participant Population

This study involved 392 participants with upper extremity dysfunction following stroke, who exhibited significant heterogeneity in their baseline functional status. Although all participants had upper extremity dysfunction, their baseline severity varied considerably, which may influence the interpretation of the study’s findings. Most studies included participants with mild to moderate impairment, who typically demonstrated some loss of motor function but still retained significant recovery potential, likely making them more responsive to interventions [25,29]. In contrast, participants with severe impairment, particularly those with baseline FMA-UE scores below 30, tend to have more complex rehabilitation needs [31]. For these individuals, the recovery process is generally slower, and intervention effects may be less pronounced.

This variation suggests that when assessing treatment outcomes, it is crucial to consider the functional status of participants at baseline, as treatment responses may differ across severity levels. Future research could refine participant stratification by standardizing baseline assessments and tailoring interventions based on varying degrees of impairment, thus more accurately evaluating treatment outcomes for different subgroups. In addition, interventions for those with severe functional impairments may need to be more intensive and personalized to accommodate their more complex rehabilitation requirements. Overall, the heterogeneity of the population in this study not only reflects diverse responses to rehabilitation but also provides important insights for future research design, particularly in terms of stratified approaches and standardized evaluations.

### Influence of System Type

The findings of this systematic review highlight that the effectiveness of home-based VR training in upper extremity rehabilitation after stroke is influenced by the type of VR system used. Customized VR systems, specifically designed for patients, offer advantages in treating both mild and severe impairments by adjusting the intensity and complexity of exercises to individual needs [33,34]. These systems have been shown to be more versatile and effective for a wider range of functional levels, including those with moderate to vigorous dysfunction, as they provide more tailored and adaptable interventions [33,35]. On the other hand, commercial VR systems, designed primarily for healthy individuals tend to be more suitable for patients with mild functional impairments, as they are often not flexible enough to accommodate the specific needs of more patients with severe impairments [36]. This discrepancy highlights the importance of customizing VR interventions to fit patient characteristics, particularly in clinical settings where maximizing the therapeutic effect is essential.

While commercial VR systems offer benefits in terms of accessibility and cost, their application in more severely impaired populations may be limited due to their higher functional demands [37,38]. Future studies should explore how to optimize commercial VR systems to expand their scope of application and validate the long-term efficacy of these interventions in patients with different levels of function. How to further enhance the personalization and sustainability of VR training in the home environment should be studied to improve its broad application in various rehabilitation scenarios.

### Influence of Intervention Intensity and Frequency

Training intensity and duration are important factors affecting the effect of VR intervention. Most studies have set the duration of the intervention between 20 and 60 minutes, and the effect varies depending on the type of human-computer interaction. Some studies have shown that intensive VR exercises, such as rapid continuous extension and contraction of the arm training, can improve the training intensity in a short period, but too long a period may cause patient fatigue and reduce participation and effect [39]. In contrast, decentralized exercises, such as VR training that simulates activities of daily living, are generally more acceptable to patients due to the familiarity of the task and can maintain a longer training time and higher compliance [25]. VR interventions with different forms of exercise significantly affected the duration of the intervention and patient acceptance. Daily activity simulation VR training can keep patients engaged for a longer period because such tasks are close to the needs of real life [40]. However, when high-intensity and monotonous intensive VR training is too long, the enthusiasm of patients may be reduced due to fatigue and repeatability [30,32]. Therefore, it is important to choose the right form of exercise and duration of intervention to balance the training intensity with the patient’s acceptance and maximize the intervention effect.

### Statistical Power and Study Heterogeneity

The small sample sizes in most studies may limit the statistical power and precision of effect estimates. The heterogeneity in study design, patient population, intervention protocols, and VR system types likely influences the observed treatment effects, making it difficult to generalize the findings. Despite this, positive trends across studies suggest that VR interventions hold promise, particularly when tailored to individual patient needs. Future research should aim to improve statistical power by increasing sample sizes and focusing on more homogeneous populations. Refining study designs to account for these variables and incorporating stratified analyses will help better assess the true impact of VR interventions across different subgroups.

### Limitations

However, there are some limitations to this study. First, heterogeneity in study design, patient population, and VR intervention protocols makes it difficult to draw consistent conclusions about the optimal characteristics of home VR training. Second, most studies had small sample sizes, inconsistent outcome measures, and lacked long-term follow-up, which reduced the reliability of conclusions and the assessment of the sustainability of intervention effects. In addition, no meta-analysis was performed due to the qualitative nature of this review, limiting insights into effect sizes and intervention efficacy. Furthermore, while the PEDro scale was used for quality assessment, the inability to blind patients and therapists in the included studies may have introduced bias and reduced the reliability of the findings. Finally, the search was limited to English-language publications, which may have introduced language bias and excluded relevant studies in other languages.

### Future Work

Future research should focus on increasing sample sizes and improving statistical power to enhance the precision and generalizability of findings. Studies should include more homogeneous participant populations to minimize baseline variability and improve the accuracy of treatment effect assessments. In addition, exploring the impact of different VR system types, intervention protocols, and their long-term effects on recovery will be crucial. Finally, standardizing outcome measures, incorporating stratified analyses, and conducting large-scale, multicenter trials with robust designs will further strengthen the evidence base.

### Conclusions

This systematic review shows that home-based VR training has a certain effect on upper extremity rehabilitation of patients with stroke, especially in patients with different degrees of dysfunction. However, challenges such as study heterogeneity, small sample sizes, inconsistent outcome measures, and limited long-term follow-up data have affected the reliability and comparability of the current findings. With the development of the technology and the popularity of applications, larger randomized controlled studies should be conducted in the future, extending follow-up time and exploring how VR technology can be integrated to support comprehensive rehabilitation programs. Home VR rehabilitation has great prospects, but its application needs to be centered on the needs of patients, combined with sustainable, personalized technology, and support services to achieve the best recovery results.

## Appendix

Multimedia Appendix 1 PRISMA_2020_checklist.

Multimedia Appendix 2 Search strategy.

## Abbreviations

ARAT: Action Research Arm Test
BBT: Box and Block Test
FMA-UE: Fugl-Meyer Assessment upper extremity section
GRASP: Glove Rehabilitation Application for Stroke Patients
NHPT: Nine‐Hole Peg Test
PEDro: Physiotherapy Evidence Database
PPT: Purdue Peg Test
PRISMA: Preferred Reporting Items for Systematic Reviews and Meta-Analysis
PROSPERO: International Prospective Register of Systematic Reviews
RCT: randomized controlled trial
VERGE: Virtual Environment for Rehabilitative Gaming Exercises
VR: virtual reality
WMFT: Wolf Motor Function Test
