# Supplementary material for: Effect of Home-Based Virtual Reality Training on Upper Extremity Recovery in Patients With Stroke: Systematic Review
Source: J Med Internet Res. 2025 Apr 4;27:e69003. doi: 10.2196/69003 (PMC12008694; doi:10.2196/69003)
Supplement: Multimedia Appendix 2 [file jmir_v27i1e69003_app2.docx]

Table 1. Search Strategy From Database Inception to June 30,2024

| Database | Search term | Results |
| --- | --- | --- |
| PubMed | (virtual reality OR VR OR video game OR telerehabilitation OR gamification OR exergame OR virtual environment) AND stroke AND upper extremity AND (home OR community) | 170 |
| Web of Science | TS=((virtual reality OR VR OR video game OR telerehabilitation OR gamification OR exergame OR virtual environment) AND stroke AND upper extremity AND (home OR community)) | 231 |
| Scoups | TITLE-ABS-KEY ( ( "virtual reality" OR "VR" OR "video game" OR "telerehabilitation" OR "gamification" OR "exergame" OR "virtual environment" ) AND "stroke" AND "upper extremity" AND ( "home" OR "community" ) ) | 194 |
| CINAHL Ultimate | (virtual reality OR VR OR video game OR telerehabilitation OR gamification OR exergame OR virtual environment) AND stroke AND upper extremity AND (home OR community) | 80 |
| Total |  | 675 |
